# Immune reconstitution and tolerance-inducing therapies as promising therapeutic approaches in multiple sclerosis

**DOI:** 10.1007/s10072-026-09328-2

**Published:** 2026-08-18

**Authors:** Alireza Beheshti Maal, Mandana Kazem Arki, Seyed Massood Nabavi, Mostafa Rezaei-Tavirani, Elham Rismani, Massoud Vosough, Gholamreza Azizi, Nikoo Hossein-Khannazer

**Affiliations:** 1 Department of Regenerative Medicine, Cell Science Research Center, Royan Institute for Stem Cell Biology and Technology, ACECR, Tehran, Iran; 2 Gastroenterology and Liver Diseases Research Center, Research Institute for Gastroenterology and Liver Diseases, Shahid Beheshti University of Medical Sciences, Tehran, Iran; 3 Proteomics Research Center, Faculty of Paramedical Sciences, Shahid Beheshti University of Medical Sciences, Tehran, Iran; 4 Molecular Medicine Department, Biotechnology Research Center (BRC), Pasteur Institute of Iran, Tehran, Iran; 5 Experimental Cancer Medicine, Institution for Laboratory Medicine, Karolinska Institute, Stockholm, Sweden; 6 Non-Communicable Diseases Research Center, Alborz University of Medical Sciences, Karaj, Iran; 7 Department of Neurology, Thomas Jefferson University, Philadelphia, PA USA; 8 Department of Tissue Engineering and Applied Cell Sciences, School of Advanced Technologies in Medicine, Shahid Beheshti University of Medical Sciences, Tehran, Iran

**Keywords:** Immune reconstitution therapy, Multiple sclerosis, Immunotherapy, Disease-modifying treatment, Immune tolerance

## Abstract

**Objectives:**

Multiple sclerosis (MS) is a complex, progressive neurodegenerative autoimmune disease and a major cause of neurological disability worldwide. MS affects approximately 2.8-3 million people, predominantly presenting as relapsing–remitting MS (RRMS) that frequently converts to secondary progressive disease, while effective options for progressive phenotypes remain limited.

**Methods:**

This review reframes MS immunotherapy through a tolerance-centric paradigm, distinguishing continuous maintenance disease-modifying therapies (DMTs) from immune reconstitution therapies (IRTs) and emerging antigen-specific tolerance strategies.

**Results:**

Classical immune reconstitution therapies, including alemtuzumab, cladribine, and autologous hematopoietic stem cell transplantation (aHSCT), have demonstrated durable disease control and prolonged periods of no evidence of disease activity (NEDA) in appropriately selected patients. This is accomplished through a finite course of lymphocyte depletion followed by qualitative immune repopulation that favors tolerogenic regulatory T (Treg) and regulatory B (Breg) cells over pathogenic Th1/Th17 clones. In contrast, maintenance DMTs (interferons, sphingosine-1-phosphate modulators, anti-CD20 monoclonals, natalizumab) suppress inflammation activity during continuous administration but lack durable immune reset. Building on IRT principles, next-generation cell-based therapies (tolerogenic dendritic cells (tolDCs), autologous Tregs, and mesenchymal stromal cells/extracellular vesicles (MSCs/EVs)), aim to induce precision, antigen-specific tolerance while minimizing systemic immunosuppression. These approaches hold promise for overcoming the limitations of chronic immunosuppression, providing durable disease control, and improving long-term patient outcomes.

**Discussion:**

Collectively, immune reconstitution and tolerance-inducing therapies represent an emerging shift from lifelong disease control toward durable immune resetting and the possibility of sustained drug-free remission in MS.

## Introduction

Multiple Sclerosis (MS) is a chronic, central nervous system (CNS) autoimmune disease in which axonal and neuronal demyelination and degeneration occurs [1, 2]. It is estimated that a total of 2.8-3 million people live with MS worldwide (35.9 per 100,000 population) [3, 4]. The mean age of diagnosis is approximately 30–32 years, with a female predominance of ~ 2–3:1 [5–7]. MS manifests primarily as relapsing-remitting MS (RRMS, ~ 85% of cases), progressing to secondary progressive MS (SPMS) in many, or primary progressive MS (PPMS, ~ 15%) from onset [8–10]. These demographics underscore the urgent need for therapies addressing both relapsing inflammation and progressive neurodegeneration.

MS immunopathogenesis centers on adaptive and innate immune dysregulation driving CNS inflammation, demyelination, and neurodegeneration [11]. Autoreactive CD4 + T cells (particularly IFN-γ-producing Th1 and IL-17/ granulocyte-macrophage colony stimulating factor (GM-CSF)-producing Th17 subsets) initiate pathology by disrupting the blood-brain barrier (BBB) via cytokines (IL-17, TNF-α) and adhesion molecules (VLA-4, LFA-1), enabling infiltration alongside pathogenic CD8 + T cells and B cells that promote antigen presentation and autoantibody production [12–14]. Within the CNS, activated microglia and astrocytes amplify neuroinflammation through TNF-α, IL-6, IL-1β, and ROS, while regulatory T cells (Tregs) and Bregs fail to restore tolerance due to impaired FOXP3/TGF-β signaling and Th17/Treg imbalance [9, 12, 15–17].

These insights have catalyzed the development of two therapeutic frameworks: continuous immunomodulation targeting pathogenic pathways (for example, S1P modulators that trap lymphocytes and anti-CD20 therapies that deplete B cells) versus immune reconstitution therapies (such as alemtuzumab, cladribine, and autologous haematopoietic stem cell transplantation (aHSCT)) that temporarily eliminate autoreactive clones. This eradication facilitates a qualitative repopulation with tolerogenic Tregs/Bregs and sustained remission [13, 18, 19]. In this review, we outline the immunopathogenesis of MS relevant to reconstitution strategies, reframe current disease-modifying therapies (DMTs) based on the sustainability of immune reset, and highlight emerging tolerance therapies, including tolerogenic dendritic cells (tolDCs), regulatory T cells (Tregs), and mesenchymal stem cells (MSCs), aimed at achieving long-term remission.

## Immunopathogenesis: therapeutic targets for immune reconstitution

### Adaptive immunity

Inflammation and demyelination, hallmarks across MS stages, are driven by proinflammatory cytokines/chemokines that disrupt the BBB and exacerbate autoimmunity [9]. Tight junction proteins (occludin, claudin-5, ZO-1) degrade under inflammatory assault, upregulating VCAM-1/ICAM-1 on endothelium to facilitate T/B cell extravasation [20, 21].

**Fig. 1:**
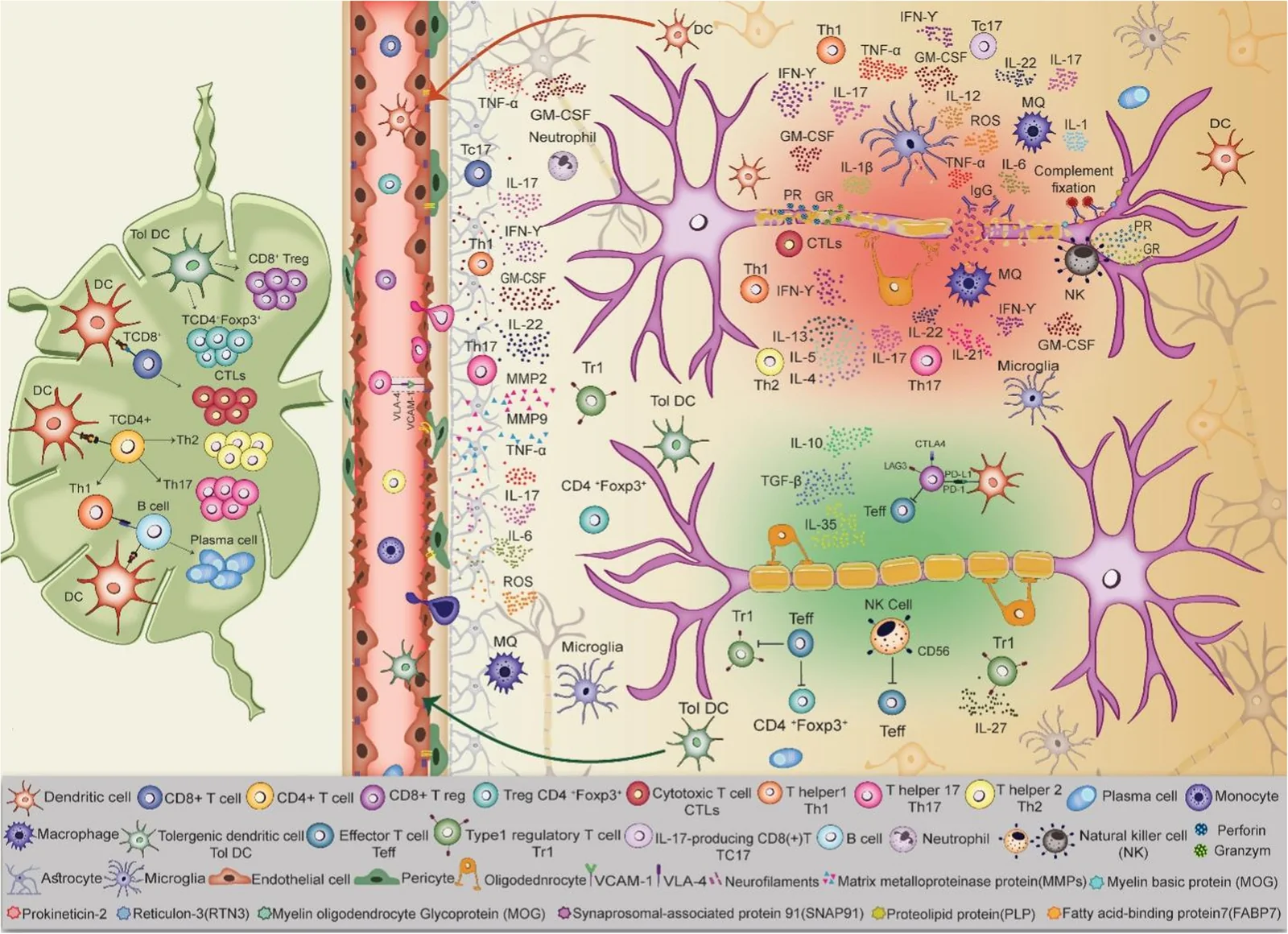
Cellular and molecular mechanism of initiation and progression of multiple sclerosis. This schematic illustrates the complex interplay between innate and adaptive immune cells contributing to central nervous system (CNS) inflammation and demyelination in multiple sclerosis (MS). In the periphery (left), dendritic cells (DCs) activate autoreactive CD4⁺ T cells, which differentiate into pro-inflammatory Th1 and Th17 subsets, as well as CD8⁺ cytotoxic T lymphocytes (CTLs) and antibody-producing B cells. These activated immune cells migrate across the blood–brain barrier (BBB) via adhesion molecules such as VLA-4 and VCAM-1. Within the CNS (right), these cells release inflammatory cytokines (e.g., IFN-γ, IL-17, GM-CSF) that activate resident microglia and astrocytes, amplifying neuroinflammation, matrix degradation, oligodendrocyte loss, and axonal injury. Regulatory populations, including CD4⁺Foxp3⁺ Treg cells, Tr1 cells, tolerogenic DCs, and M2 macrophages, contribute to immune tolerance and neuroprotection. The figure also includes recently identified MS-associated autoantigens such as: Synaptosomal-associated protein 91 (SNAP91), Prokineticin-2 (PROK2), Reticulon-3 (RTN3), and Fatty acid-binding protein 7 (FABP7) and highlights the balance between pro and anti-inflammatory mechanisms that dictate disease progression

MS is a complex immune-mediated disease characterized by dysregulated interactions among autoreactive CD4⁺ and CD8⁺ T cells, B cells, antigen-presenting cells, and resident CNS immune cells, resulting in chronic inflammation, demyelination, and neurodegeneration (Fig. 1). Autoreactive Th1 (IFN-γ+) and Th17 (IL-17+/GM-CSF+) cells target myelin antigens (myelin oligodendrocyte glycoprotein (MOG), myelin basic protein (MBP), and proteolipid protein (PLP)), with 2022–2025 discoveries of novel autoantigens (synaptosomal-associated protein 91 (SNAP91), prokineticin-2 (PROK2), reticulon-3 (RTN3) and, fatty acid–binding protein 7 (FABP7)) implicating broader epitope spreading [22, 23]. Th17 cells predominate in relapses, recruiting myeloid cells via IL-17/IL-22/GM-CSF and resisting Treg suppression [24, 25]. CD8 + T cells, via cross-presentation, deliver perforin/granzyme to oligodendrocytes/axons; IL-17-secreting Tc17 subsets amplify chronic lesions [26, 27].

Current evidence indicates that both T cells and B cells play complementary and interconnected roles in MS pathogenesis. B cells sustain pathology beyond antibodies: they present antigens to T cells, secrete TNF-α/GM-CSF/lymphotoxin, and harbor clonally expanded autoreactive pools that may persist for years before clinical disease onset. Recent studies have also identified predictive autoantibody signatures that precede symptom onset, highlighting the potential of humoral biomarkers for early disease detection [28, 29]. Recent anti-CD20 data reveal CD20dim T cells (acquired via trogocytosis) as pro-inflammatory effectors correlating with MRI activity, underscoring B-T crosstalk [30].

It is worth mentioning that Epstein-Barr virus (EBV) infection has emerged as a prominent risk factor that exert its effect via immune cell. EBV persists predominantly within memory B cells and may promote autoreactive immune responses through chronic antigenic stimulation and molecular mimicry [31]. The demonstration that EBV infection precedes nearly all cases of MS has reinforced the concept that B cells represent not only important immune effectors but also potential reservoirs linking environmental exposure with autoimmune disease initiation [32]. Beyond its role as a viral reservoir in memory B cells, primary evidence, now supported by multiple independent studies, confirms that EBV initiates neuroinflammation through molecular mimicry at the T-cell level, specifically involving cross-reactive CD4 + T-cell responses against the host protein Anoctamin-2 (ANO2) in approximately 57% of patients [33–35]. Furthermore, EBV-infected B cells can genetically reprogram themselves to present ‘hidden’ myelin basic protein (MBP) peptides that have escaped central thymic tolerance, thereby activating a persistent pool of autoreactive T cells [36]. This evolving understanding provides additional biological support for B-cell-targeted immune reconstitution strategies.

In contrast, Th2 cells (IL-4/IL-13), CD4 + CD25+FOXP3 + Tregs (IL-10/TGF-β), CD8 + FOXP3+ Tregs, Bregs, and Type 1 regulatory (Tr1) cells exhibit numeric and functional deficiencies. For instance, the plasticity of FOXP3-low IL-17 + Tregs permit unregulated autoreactivity [37–39]. This imbalance, favoring effectors over regulators, represents the key therapeutic insight for immune reconstitution strategies that deplete pathogenic clones while fostering tolerogenic repopulation [13, 40].

### Innate immunity

Although adaptive immune responses play a major role in MS, innate immune cells also have a pivotal role in disease pathogenesis (Fig. 1). CNS-resident glial cells, including microglia and astrocytes, shift to proinflammatory states, secreting TNF-α, IL-1β, IL-6, ROS, and chemokines (CCL2/3/5, CXCL10) that recruit adaptive and drive oligodendrocyte death [41, 42]. Microglia phagocytose myelin, present antigens, and polarize astrocytes to A1-neurotoxic phenotypes; M1-like macrophages predominate early, yielding to M2-reparative in remission [43, 44].

DCs as the main APCs, are the main modulators of self-tolerance. DCs present antigens to naïve T cells in lymph nodes, promote T cell polarization toward different sub-populations (Th1,2,17, reg) and determine the immune response outcome. It was shown that MS patients had defects in monocyte to DC differentiation and irregular DC distribution. The expression of CD80, CD86, and CD83 co-stimulatory molecules increases and leading to a dysregulated balance of pro-inflammatory and anti-inflammatory cytokine profiles in these patients [45, 46]. This dysregulated priming perpetuates peripheral autoreactivity, a target for tolerogenic DC (tolDC) therapies.

Natural killer (NK) cells are powerful innate immunity cytotoxic cells that have both positive and negative roles in MS pathogenesis. It was shown that CD56 ^bright^ NK cells had immunomodulatory effects and inhibit effector T cells by granzyme B production whereas, CD56^dim^ NK cells have more cytotoxic effects. However, the total number of NK cells was reduced in MS patients compared with healthy individuals and correlated with the formation of lesions and relapse. In addition, following immunomodulatory therapy, the number of NK cells increased and correlated with treatment response [47].

Inflammatory macrophages (M1) like activated microglia cells initiate inflammatory reactions in the early stages of the MS disease. Infiltrated M1 macrophages promote inflammatory responses and antigen presentation through the production of IL-1, IL-6, TNF-α, IL-12 and, NO in MS lesions. Accumulation of M1 cells increases neuron damage and demyelination while the M2 (anti-inflammatory) phenotype inhibits inflammation and enhances remyelination and repair [48]. The balance between M1 and M2 phenotypes which is crucial to maintaining tolerance is disrupted and M2/M1 phenotype switching occurs during MS pathogenesis [43].

Recent research indicates that neutrophils also play an important role in MS disease. The number and inflammatory functions of neutrophils markedly upregulated in MS patients who experience the early phase of relapse and new lesions formation. Neutrophils secret large amounts of IL-1β, GM-CSF which generate a positive feedback loop for excessive inflammation. Neutrophil derived matrix metalloproteinase protein 2,9 (MMP2,9), myeloperoxidase (MPO) and ROS promote BBB breakdown [49]. Additionally, an increased neutrophil-to-lymphocyte ratio (NLR) index is associated with a higher risk of relapse within two years and may be a useful biomarker for follow up screenings [50].

These innate effectors form a feedforward loop characterized by adaptive, diminishing tolerance. Notably, post- immune reconstitution therapies (IRTs) immune reconstitution indicates qualitative shifts including reduced M1 glia, expanded tolDCs, and NK/Treg recovery, highlighting the role of innate mechanisms in sustaining remission [51–53]. Overall, due to the role of immune responses in MS pathogenesis, it is considered that targeting pathogenic immune cells and induction of peripheral tolerance could be an effective therapeutic approach.

## Principles of multiple sclerosis immunotherapies

**Fig. 2:**
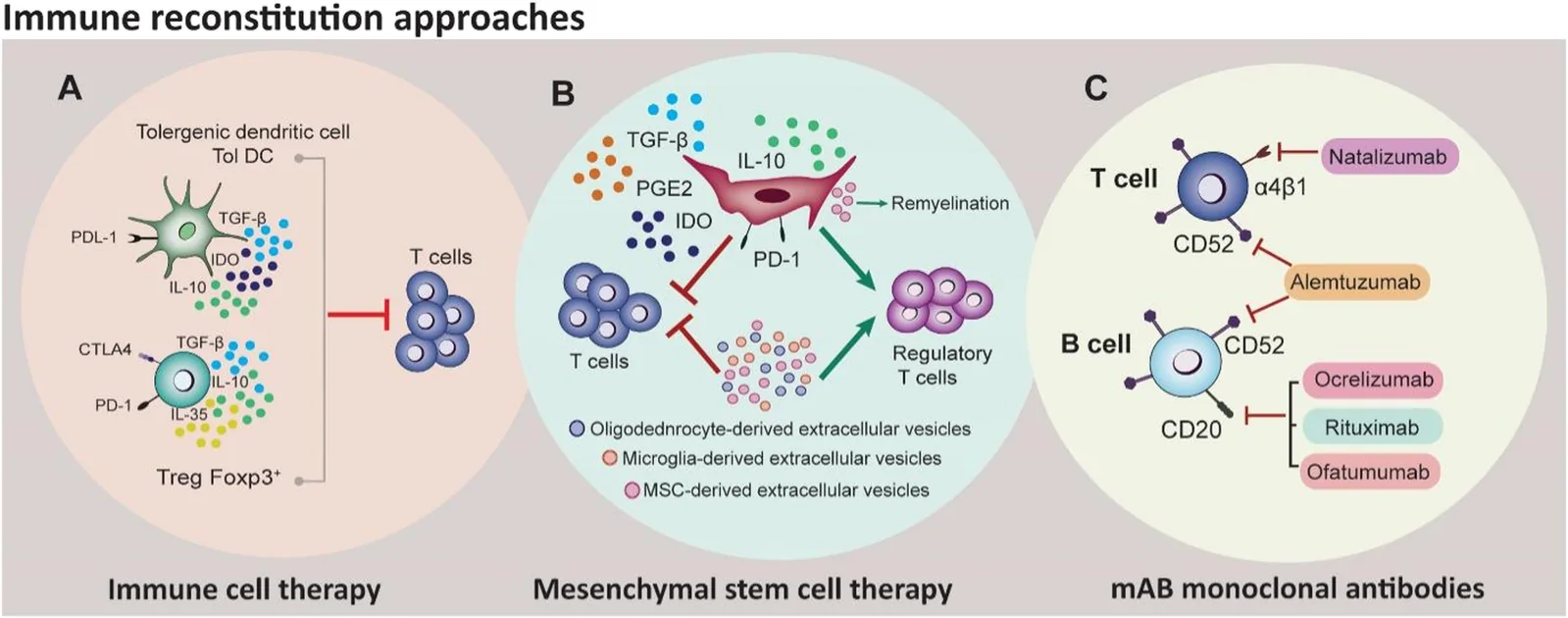
Immune Reconstitution Approaches in Multiple Sclerosis. Three major therapeutic strategies for immune reconstitution in MS (**a**) Immune cell therapy: TolDCs and Treg cells suppress autoreactive T cells via anti-inflammatory cytokines (IL-10, TGF-β), immune checkpoints (PD-L1, PD-1, CTLA-4), and enzymes such as IDO (**b**) Mesenchymal stem cell therapy: MSCs and their extracellular vesicles (EVs) modulate immune responses through secretion of immunosuppressive and neuroprotective factors (TGF-β, PGE2, IDO), inhibit pathogenic T cells, induce regulatory T cells, and promote remyelination (**c**) Monoclonal antibody therapy: Targeted depletion of T and B cells using natalizumab (α4β1 integrin), alemtuzumab (CD52), and anti-CD20 antibodies (ocrelizumab, rituximab, ofatumumab) effectively reduce disease activity by eliminating autoreactive lymphocytes

MS phenotypes follow the same pathophysiology and inflammation and tissue damage are present in all patients. Highly effective DMTs have revolutionized RRMS management, however treatment of progressive forms (SPMS/PPMS) remain challenging, with few options beyond ocrelizumab. Current DMTs for MS disease are divided into two strategic paradigms of continuous maintenance therapies and immune reconstitution therapies (IRTs), based on mechanism and durability (Table 1; Fig. 2). Continuous maintenance DMTs require persistent management to ensure sustained immune modulation (platform therapies, oral small molecules, continuous high-efficacy monoclonals). In contrast, IRTs involve limited treatments that reduce pathogenic immune cells, enabling qualitative immune repopulation and potential drug-free remission.

**Table 1:** Current disease-modifying therapies for multiple sclerosis and their mechanisms of action. IFN-β, interferon beta; CIS, clinically isolated syndrome; RRMS, relapsing remitting MS; PPMS, primary progressive MS; SPMS, secondary progressive MS

Drug Name	Year of approval	Mechanism of action	Route of administration	MS type	Side effects
IFN-β					
IFN-β 1b	1993	Inhibition of lymphocyte proliferation and antigen expression, regulation of anti- inflammatory phenotypic cytokinesis products in the circulatory system and CNS, inhibition of T cell matrix metalloproteinase activity, and reduction of inflammatory T cell migration	Subcutaneous	CIS and RRMS	Injection-site inflammation, flu-like symptoms, rhinitis, and headache
IFN-β 1a	2002	Inhibition of lymphocyte proliferation and antigen expression, regulation of anti- inflammatory phenotypic cytokinesis products in the circulatory system and CNS, inhibition of T cell matrix metalloproteinase activity, and reduction of inflammatory T cell migration	Subcutaneous	CIS and RRMS	Injection-site inflammation, flu-like symptoms, rhinitis, and headache
PeglFN β-la	2014	Enhancement of the production of anti-inflammatory cytokines such as interleukin (IL) 4, IL-5, IL-10, IL-13, IL-27, and transforming growth factor beta, as well as the suppression of proinflammatory cytokines such as IL-17, IFNγ, and tumor necrosis factor alpha.	Subcutaneous	CIS and RRMS	Injection-site inflammation, flu-like symptoms, Lymphopenia
Glatiramer Acetate					
Glatiramer Acetate	1996	Not completely understood. Activation of T cells and induction of Th2 cell production.	Subcutaneous	RRMS	Injection-site reactions, flushing, chest tightness, dyspnea, palpitations, anxiety after injection, and less commonly, lipoatrophy
Mitoxantrone					
Mitoxantrone	2000	Inhibits cellular DNA replication, transcription, and repair.	Intravenous	RRMS and SPMS	Cardiotoxicity, hair loss, constipation, and abnormal liver function
Sphingosine 1-phosphate (S1P) antagonists					
Fingolimod	2010	Inhibit migration of lymphocytes from secondary lymphoid organ to CNS.	Oral	RRMS	Bradycardia, atrioventricular conduction block, macular edema, elevated liver- enzyme levels, and mild hypertension
Ozanimod	2020	Inhibit migration of lymphocytes from secondary lymphoid organ to CNS.	Oral	CIS and RRMS	First-dose bradycardia and conduction block, headache and elevated liver aminotransferase
Siponimod	2019	Inhibit migration of lymphocytes from secondary lymphoid organ to CNS.	Oral	CIS, RRMS, active SPMS	Headache, nasopharyngitis, urinary tract infection, and falls
Teriflunomide					
Teriflunomide	2012	Reduction of the replication capabilities of immune cell, limit the immune response	Oral	RRMS	Headache, diarrhea, nausea, alopecia, and increase in hepatic alanine transferase
Dimethyl fumarate					
Dimethyl fumarate and diroximel Fumarate	2013	Activation of the nuclear factor (erythroid-derived 2)–like 2 (Nrf2) pathway and Nrf2-independent pathways	Oral	RRMS	Flushing, diarrhea, nausea, upper abdominal pain, decreased lymphocyte counts, elevated liver aminotransferase and rarely PML
Cladribine					
Cladribine	2019	Not completely understood. Accumulation in lymphocyte population, inducing apoptosis in lymphocytes	Oral	RRMS	lymphopenia, with an increased risk of herpes zoster virus infection

### Continuous maintenance disease modifying therapies

DMTs immunological targets, effects and mechanism of action are greatly varied and based on their efficacy they are classified into low-efficacy, moderate-efficacy, and high-efficacy groups. The final goal of using DMTs is reaching optimal therapeutic response known as no evident disease activity (NEDA). In spite of DMTs success in reducing relapse rate and MRI activity, accumulating evidence indicates that many patients continue to experience disability progression in the absence of overt clinical relapses, a phenomenon now recognized as progression independent of relapse activity (PIRA) [54]. PIRA has emerged as a major mechanism of long-term disability accumulation across the MS disease spectrum, including the early stages of relapsing-remitting MS. Recurrent PIRA events contribute substantially to irreversible neurological worsening, accounting for a large proportion of disability accumulation in relapsing MS and becoming the predominant driver of progression in secondary and primary progressive MS [55, 56]. Consequently, the importance of initiation of highly effective therapies early in the course of MS which enhance the control of the relapse and progression is highlighted [10, 57]. In this approach, which involves immunosuppressive or immunomodulatory modalities, uninterrupted and continuous drug administration is essential to achieve sustained inflammation suppression and disease remission. Immunosuppressant drugs exert their effects by broadly inhibiting immune system activity for the duration of their use. However, prolonged administration of these agents is often associated with adverse effects, including lymphopenia, an elevated risk of infectious diseases, and an increased likelihood of cancer development [53].

#### Platform therapies (low-modest efficacy)

##### Interferon Beta (IFN-β)

Interferon beta (IFN-β) was the first drug approved for RRMS. IFN-β inhibits lymphocyte proliferation, expansion, migration and, activation. IFN-β can modulate cytokine production toward an anti-inflammatory profile, reduce leukocyte trafficking across the blood-brain barrier, and increase anti-inflammatory mediators in peripheral blood and the CNS [58, 59].

Treatment with IFN-β decreases the relapse rates and the number of lesions. There are four approved preparations of the drug: IFN-β-1b and IFN-β-1a which are injected subcutaneously, intramuscular IFN-β-1a which is injected once a week and, Peginterferon beta-1a (pegylated formulation). Peginterferon beta-1a has a longer half-life compared to other formulations and reduces the number of administrations. Subcutaneous administration of Peginterferon beta-1a decreased inflammatory responses and had beneficial effects on RRMS patients [60].

##### Glatiramer Acetate (GA)

Glatiramer Acetate (GA) is an immunomodulating amino acid copolymer and the second medication that received FDA approval for MS. GA acts via Th1 cells shift to Th2 phenotype and induces Th2 and Treg cells. Th2 cells produce IL-4, TGF-β and, IL-10 anti-inflammatory cytokines. It modestly reduces relapse rate, has a modest effect on measures of disease severity and generally has similar efficacy compared to IFN-β in RRMS [61]. Its relatively safe and common side effects include: injection-site reactions, flushing, injection anxiety, lipoatrophy and, inflammatory skin reactions. However, side effects are not associated with immunological responses injection [62]. In addition to these side effects, daily injection requirements of GA can negatively influence treatment adherence.

#### Oral Maintenance Therapies (moderate efficacy)

##### Sphingosine 1-phosphate (S1P) receptor modulators

Sphingosine 1-phosphate (S1P) receptor modulators bind to S1P receptors on lymphocytes and cause internalization and degradation of S1P receptors which results in trapping of lymphocytes in the secondary lymphoid tissues [63]. S1P receptor modulators include fingolimod (FTY), Siponimod, and ozanimod. Fingolimod also reduces the expression of S1PR on the oligodendrocytes and neurons, and plays role in neuroprotection and repair [64]. Fingolimod was the first oral agent approved for MS in 2010. Trials have shown that FTY is effective in recurrent MS and can reduce the burden of injection. Head-to-head studies showed that fingolimod was well tolerated and superior to intramuscular IFN-β [65].

Ozanimod has gained FDA approval in 2020. It has a similar mechanism of action compared to FTY with a shorter half-life than fingolimod, and lymphocyte recovery is faster after its discontinuation [66]. More recently, a selective S1P modulator called Siponimod has shown promising results in treating patients with active SPMS. It has been proven that Siponimod inhibited disability progression [67].

##### Teriflunomide

Teriflunomide is the 2nd oral agent approved for RRMS. It is an active metabolite of leflunomide, used in rheumatoid arthritis treatment. Teriflunomide is an inhibitor of pyrimidine synthesis via dihydroorotate dehydrogenase, which reduces the proliferation of immune cells, limits immune responses, and reduces MS activity [68]. It is indicated for the treatment or relief of recurrent MS. Common side effects of the drug include: headache, diarrhea, alopecia, and elevate alanine aminotransferase levels [69].

##### Bruton tyrosine kinase inhibitors (BTKis)

Bruton tyrosine kinase inhibitors (BTKis) are among the newest therapeutic approaches proposed in treatment of MS. These drugs target both peripheral adaptive immunity (B cells) and CNS-compartmentalized innate immunity (microglia and macrophages) [70]. While early results of Evobrutinib and Tolebrutinib in RMS were promising, later stage results of evolution RMS1 & 2 and GEMINI 1 & 2 for Evobrutinib and Tolebrutinib respectively, showed moderate efficacy in preventing disability progression and generally fell short of high-efficacy biologic therapies in reducing relapse rates [71, 72]. On the other hand, promising results were observed in the HERCULES trial. In this phase 3 randomized, placebo-controlled study, Tolebrutinib helped patients with non-relapsing SPMS by significantly slowing the accumulation of disability and reducing markers of chronic inflammation [73]. However, in primary progressive MS, the PERSEUS trial (trial registration number; NCT04458051) failed to meet its primary endpoint of delaying time to six-month composite confirmed disability progression, and the sponsor subsequently announced that it would not pursue regulatory approval for this indication (https://www.sanofi.com/en/media-room/press-releases/2025/2025-12-15-06-05-00-3205094). It is worth mentioning that in a recently network meta-analysis of therapeutic strategies for SPMS reported that Tolebrutinib and siponimod were among the most effective agents in delaying disability progression compared with other studied treatments [74]. These findings indicate that BTK inhibition may have differential effects across progressive MS phenotypes. Notably, Fenebrutinib has provided the first positive phase 3 signal for a BTK inhibitor in progressive disease: in FENtrepid (trial registration number; NCT04544449), it met its primary endpoint by demonstrating non-inferiority to Ocrelizumab for time to composite confirmed disability progression, and in FENhance 2 it also met its primary endpoint in relapsing MS by demonstrating superiority to teriflunomide. Together, these results support the dual B-cell and microglial rationale underlying BTK inhibition in progressive disease. Detailed peer-reviewed results are awaited (https://www.roche.com/investors/updates/inv-update-2025-11-10). Despite these benefits, hepatotoxicity has emerged as a class concern, necessitating frequent liver function monitoring during the initial months of therapy [73].

##### Dimethyl fumarate

Dimethyl fumarate (DMF) is another oral medication approved by the FDA in 2013. It exerts immunomodulatory effects through activation of the nuclear factor (erythroid-derived 2)–like 2 (Nrf2) pathway and Nrf2-independent pathways [75]. It was also demonstrated that DMF could induce Nrf2-dependent antioxidant genes in MS patients. In phase III clinical trials, patients receiving dimethyl fumarate had a significant reduction in annualized relapse rate (ARR) and reduced both MRI and clinical disease activity. DMF is generally well-tolerated but has some adverse events including diarrhea, nausea, flushing, and abdominal pain. Diroximel fumarate (DRF) is recently approved for MS, and is closely related to dimethyl fumarate and has the same pharmacodynamics. In a phase III trial, it was demonstrated that DRF improved gastrointestinal tolerability with less gastrointestinal side effects [76].

#### Continuous high-efficacy monoclonals

##### Natalizumab

Natalizumab is a humanized monoclonal antibody against α4β1 integrin, an adhesion molecule involved in lymphocyte transmigration into the CNS. Natalizumab blocks the migration of the active lymphocytes to CNS by inhibiting their adherence and interaction with endothelium. Natalizumab is highly effective in decline relapses and limit disease progression in RRMS patients compared to IFN-β [77]. It is administered intravenously every 4 weeks, is well tolerated, and has a low-risk safety profile. The most major side effect of natalizumab is, progressive multifocal leukoencephalopathy (PML) which occurs in ~ 0.4% of natalizumab-treated patients [78]. AFFIRM trial showed ARR reduction 68% (0.73 to 0.23, *p* < 0.001) vs. placebo; 42% relative risk reduction in disability progression at 2 years. Real-world TOP registry (*n* = 6148) natalizumab reduced ARR from 1.99 (95% CI 1.97–2.02) at baseline to 0.15 (95% CI 0.14–0.15) during treatment, corresponding to a 92.5% reduction in annualized relapse rate [79, 80].

##### **Anti-CD20 Monoclonal antibodies (Ocrelizumab**,** Ofatumumab**,** Rituximab**,** Ublituximab)**

Monoclonal antibodies against B cells CD20 molecules could inhibit migration of these cells to CNS and subsequently reduce their activation and differentiation to plasmablasts. Ocrelizumab is a humanized anti-CD20 monoclonal antibody. Trials have demonstrated the potency of ocrelizumab in reducing relapse rates and slowing MS progression. It is administered via IV transfusion every 24 weeks. Except for Herpes zoster infection, no major adverse events were observed [81]. Recent studies on ocrelizumab administration for the progressive forms showed promising results [82]. Subsequently, ocrelizumab became the 1st approved treatment for PPMS in 2017. Earlier phase II trials of rituximab have shown a similar efficacy compared to ocrelizumab. Rituximab is widely used despite the fact that it has never been approved by any regulatory body. Because ocrelizumab is inaccessible in many countries, rituximab is used instead [83]. Ofatumumab marketed under the brand name of Kesimpta was approved by European Medicines Agency (EMA) for RRMS in 2020. It is a fully human anti-CD20 monoclonal antibody and reduce B cells population. Ofatumumab is administered subcutaneously once in each month and patients can inject it themselves. Ofatumumab has a similar efficacy profile to ocrelizumab. In phase III clinical trials the safety of ofatumumab was approved and adverse events were manageable. Ofatumumab was effective in reducing relapse rate and MS lesions and, disease activity improved in MRI. Moreover, targeting B cells with ofatumumab decreased MS progression and disease disability in RRMS individuals [84].

Ublituximab is another anti CD-20 therapy which received FDA approval in 2022 and EMA approval in 2023 [85]. The strongest direct evidence comes from the twin phase III ULTIMATE I/II trials, which enrolled 549 participants and found significantly lower relapse rates with Ublituximab than teriflunomide over about 96 weeks. Additionally, Ublituximab produced marked MRI suppression, with far fewer gadolinium-enhancing lesions and fewer new/enlarging T2 lesions than teriflunomide at 96 weeks [86]. Post hoc pooled analyses also found substantially higher NEDA rates, including 44.6% versus 12.4% over weeks 0–96 and 82.1% versus 22.5% over re-baselined weeks 24–96 [87]. Table 2 presents the current monoclonal antibodies utilized in the treatment of MS.

**Table 2:** Current and ongoing monoclonal antibodies for multiple sclerosis. IFN-β, interferon beta; CIS, clinically isolated syndrome; RRMS, relapsing remitting MS; PPMS, primary progressive MS; SPMS, secondary progressive MS; mAb, monoclonal antibodies

Monoclonal antibody	Year of approval	Mechanism of action	Route of administration	MS type	Side effects
Alemtuzumab	2014	Anti-CD52 mAb (a receptor that is present on lymphocytes, monocytes and other immune and non-immune cells)	Intravenous	RRMS	Infusion reactions secondary to cytokine release syndrome, herpetic infections, secondary autoimmunity, including thyroid disorders
Natalizumab	2004	α4β1 integrin inhibitor (blocking the adhesion of lymphocytes)	Intravenous	RRMS	Fatigue, allergic reaction and progressive multifocal leukoencephalopathy (PML)
Ocrelizumab	2017	Anti-CD20 mAb (inhibits migration of these cell to CNS and subsequently reduce activation and differentiation to immunoglobulin secreting plasmablasts)	Intravenous	RRMS and PPMS	Infusion-related reaction, nasopharyngitis, upper respiratory tract infection, headache, and urinary tract infection
Ofatumumab	2020	Anti-CD20 mAb (inhibits migration of these cell to CNS and subsequently reduce activation and differentiation to immunoglobulin secreting plasmablasts)	Subcutaneous	RRMS	Injection-related reaction, nasopharyngitis, headache, upper respiratory tract infection, and urinary tract infection
Ublituximab	2022	Anti-CD20 mAb (inhibits migration of these cell to CNS and subsequently reduce activation and differentiation to immunoglobulin secreting plasmablasts)	Intravenous	RRMS	Infusion-related reaction, Urinary tract infection, Respiratory infection, Fever
Rituximab	Not yet approved	Anti-CD20 mAb (depletion of B cells, Prolonged reduction of memory B cells)	Intravenous	RRMS	Infusion-related reaction, Infection-associated events

### Immune reconstitution therapy

Recently, immune reconstitution therapies (IRTs) have emerged as one of the most promising therapeutic strategies in MS. These approaches involve short-course treatment regimens that induce long-lasting immunological changes and sustained disease control [88]. In this approach selected immune components are eliminated through short term severe immune suppression. IRTs allow the immune system to reconstitute itself and restore lost immune tolerance. It is suggested that IRT therapies are very effective inducing long-term drug-free remission and decreasing adverse effects associated with continuous immune suppression [89].

#### Alemtuzumab

Alemtuzumab is a humanized monoclonal antibody against CD52 and approved by the FDA for RRMS. Alemtuzumab targets CD52 which highly expressed on B and T lymphocytes. It is administrated intravenously for 5 successive days. This monoclonal antibody, could effectively deplete pathogenic lymphocytes and re-establish adaptive immune systems. Studies indicated that following Alemtuzumab therapy the levels of inflammatory markers including cytokines and chemokines decreased in patients [90]. Moreover, while Th1 and Th17 cells and their relative cytokines decreased, T and B reg cell populations were induced. Trials indicated that Alemtuzumab significantly reduced relapse rate, disability, and neuron loss in MS patients. Alemtuzumab had long lasting effects to induce NEDA in RRMS patients. However, using Alemtuzumab is limited due to the severe adverse events including secondary autoimmune disorders like thyroiditis [91].

#### Cladribine (CLAD)

Oral CLAD (2-chlorodeoxyadenosine) is a synthetic deoxyadenosine analog that is approved by the FDA for RRMS disease. CLAD interferes with DNA synthesis and repair and, therefore, induces cell death mediated by caspase-3 activation. CLAD therapy decreased circulating pro-inflammatory lymphocytes especially CD19 + B cells. It could also induce sustained suppression in the memory B cell population [92]. Additionally, as a depleting medication, CLAD eliminates monocytes, macrophages and, NK cells and inhibits the influx of immune cells to the CNS. Various studies showed that CLAD is clinically efficient and significantly improved disability, reduced relapse rates, and MRI activity in MS patients compared to control. Observational cohort studies confirmed the long-term safety and effectiveness of oral CLAD in RRMS treatment [93, 94].

#### Autologous hematopoietic stem cell transplantation

Although HSC transplantation (HSCT) is largely used in hematologic disorders and malignancies; it has also demonstrated promising benefits in autoimmune diseases such as MS [95]. Studies indicate that autologous hematopoietic stem cell transplantation (aHSCT) could induce remission in MS patients and improve neurological functions in RRMS patients [96]. Moreover, these cells enhanced durable improvement in patients’ disability and inhibited disease worsening and progression [97]. AHSCs reduce autoreactive lymphocytes specifically Th17 cells and induce Treg cell populations [98]. For autologous HSC transplantation, HSCs (usually from peripheral blood) are mobilized using GM-CSF or granulocyte colony-stimulating factor (G-CSF). Afterward, harvesting and positive selection for CD34 + HSCs are done ex vivo. Finally, patients undergo HSC transplantation after ablative therapy (eliminating all autoreactive immune cells) [99]. Following aHSCT, pathogenic Th1 and Th17 cells decreased while Treg cells and naïve T cells increased. Post aHSCT immune reconstitution indicated that inflammatory cytokines and chemokines shift to anti-inflammatory profile. AHSCT is an efficient therapy for suppressing inflammation and immune reactions in aggressive RRMS patients [100]. However, its use remains limited by the need for intensive conditioning regimens, specialized transplant expertise, and the risk of treatment-related complications, including severe infections, prolonged cytopenias, infertility and, although now uncommon in experienced centers, treatment-related mortality. Therefore, careful patient selection and management in experienced multidisciplinary centers are essential to maximize benefit while minimizing procedural risk [100].

## Innovative tolerance inducing strategies in multiple sclerosis

Novel treatment approaches for multiple sclerosis aim to eliminate inflammatory immune cells, reset the immune system to promote self-tolerance, and enhance neuronal regeneration. These novel approaches include checkpoint inhibitor agonists, co-stimulatory molecules antagonists, cytokine-based therapies, vaccines (DC-based and DNA-based vaccines), and tolerance inducing cell therapies (Fig. 2) [101]. The clinical relevance of PIRA further supports the rationale for immune reconstitution and antigen-specific tolerance strategies, because continuous immunosuppression may reduce relapses yet still fail to durably prevent progression. In contrast, therapies designed to re-establish immune tolerance may more directly address the biology underlying repeated PIRA-related disability accrual [53, 56]. This section integrates classical IRTs with emerging antigen-specific tolerance approaches under a unified immune tolerance framework achieving durable remission through autoreactive clone deletion, tolerogenic skewing, and innate compartment modulation. We contrast these with maintenance DMTs via mechanistic principles (Table 3), then detail cell-based tolerance therapies representing the next therapeutic frontier.

**Table 3:** Tolerance strategy continuum

Strategy	Duration	Lymphocyte Depletion	Repertoire Change	Treg/Breg Restoration	Long-term Safety
Maintenance DMT	Continuous	None/ partial (B cells)	None	Minimal	Cumulative infections/malignancy
Classical IRT	Finite (weeks)	Deep (T/B/NK)	Naive/ tolerogenic shift	Strong (FOXP3↑, IL-10↑)	Secondary autoimmunity
Cell-based Tolerance	Single/few doses	Minimal/none	Antigen-specific anergy	Maximal (clonal expansion)	Excellent (physiologic)

### Mechanistic continuum of tolerance strategies

Classical IRTs (alemtuzumab, cladribine, aHSCT) achieve control of disease via non-specific depletion followed by qualitative repopulation. Emerging antigen-specific therapies (tolDCs, Tregs, MSC-derived EVs) target myelin-reactive clones precisely, minimizing off-target effects while amplifying shared mechanisms such as Treg/Breg expansion, Th17 suppression, and innate immune repolarization.

### Classical IRTs as tolerance inducers

Classical IRTs restore self-tolerance through profound immune repertoire reset. Alemtuzumab, targeting CD52, achieves 70–90% reduction in proinflammatory Th1/Th17 cells alongside 2–3-fold expansion of CD4 + FOXP3+ Tregs within 12 months post-depletion, with regulatory B cell (Breg) reconstitution strongly correlating with 5-year NEDA status [102]. Cladribine selectively depletes memory B cells while relatively preserving or increasing the naive B cell compartment. Furthermore, while treatment induces a sustained reduction in proinflammatory Th1 and Th17 cells, it is accompanied by a concomitant and continued decline in the absolute counts of non-follicular regulatory T cells (Tregs) throughout the second year of therapy [92]. aHSCT delivers the most comprehensive reset, rendering autoreactive TCR/BCR clones undetectable post-transplant through lymphoablation and thymic reconstitution. Meta-analyses confirm that 68% of patients experienced NEDA after aHSCT [97, 103, 104].

### Antigen-specific tolerance: next-generation IRT

One approach to achieving persistent self-tolerance and targeting the underlying mechanism of autoimmune reactions is tolerogenic inducing cell therapies. Cell-based therapies for the treatment of MS disease attract lots of attention. Cell therapies could suppress inflammatory mediators, inhibit neuron loss, and improve neural regeneration and remyelination. Various tolerogenic cell sources including immune cells and non-immune cells (mesenchymal stromal cells, MSCs) are used in cell therapies with different therapeutical approaches which finally result in peripheral tolerance induction [105]. Numerous pre-clinical and clinical studies have shown that cell-based therapies have beneficial effects on the clinical manifestations of MS disease. In this regard, we discuss cell-based treatment approaches including immune cell therapies like Treg cells and tolerogenic DCs (tolDCs), and MSCs therapies as non-immune cell transplantation in the treatment of MS.

#### Tolerogenic Dendritic Cells (tolDCs)

Targeting DCs as the most important antigen presenting cells (APCs) and the major regulators of adaptive immune responses is a promising approach in the treatment of autoimmune diseases including MS [106]. TolDCs which are characterized by the expression of anti-inflammatory cytokines (IL-10, TGF-β) and immunomodulatory molecules (iNOs, IDO, PDL1, PDL2) impeded antigen processing and presentation, inhibited effector T cells functions, induced T cell anergy and, cause apoptosis through Fas signaling pathway (Fig. 2.A). Moreover, these cells could induce the proliferation and expansion of Treg cells. Pre-clinical studies have revealed that tolDCs inhibited antigen specific autoimmune responses by inducing Treg cells and producing IL-10 and TGF-β [107–109]. TolDC therapy in animal models indicated reduced T cell proliferation and functions. TolDCs decreased disease severity and had beneficial effects in inducing peripheral tolerance [110, 111].

The safety of dexamethasone induced tolDCs (Dexa-tolDC) was investigated in an early phase Ib clinical trial in patients with MS and neuromyelitis optica. It was shown that antigen specific tolDCs were well tolerated and no severe adverse effects occurred. Immunological evaluations indicated a shift toward Th2 responses with increased IL-10 production, and reduced IFN-γ production [112]. In the subsequent peer-reviewed phase 1b trial, administration of peptide-loaded tolerogenic dendritic cells was found to be safe and feasible and patients remained clinically stable, and immunological assessment showed increased IL-10 and Tr1 responses [113]. Crucially, the identification of cross-reactive T-cell targets between EBV and the CNS, such as EBNA1 mimicry of ANO2, provides a mechanistic rationale for antigen-specific tolerance strategies, including tolDC and engineered Treg approaches, enabling selective targeting of pathogenic clones [33, 35]. In a study, clinical-grade autologous tolDCs which generated with GM-CSF, interleukin 4 (IL-4), 1α,25 dihydroxy vitamin D3 and, pulsed with myelin peptides (tolDC-VitD3), injected through intradermal/intranodal routes in MS patients. In phase I (dose-escalating phase), the safety and tolerability of the tolDC-VitD3 will be assessed and the most appropriate treatment protocol will be selected (trial registration numbers; NCT02618902 and NCT02903537) [114]. Previous in-vivo studies showed that tolDC-VitD3 had beneficial effects on EAE mice through induction of PD-1/PD-L1 pathway. Studies revealed that soluble CD83 (sCD83) molecule on DCs has immunosuppressive activity and could ameliorate disease in EAE animal model. Results of a recent DC therapy on type 1diabetes indicated that engineered tolDC encoding peptide antigen and IL-10 (DC^IL−10/Ag^) could effectively inhibit effector CD4 + and CD8 + T cells and could be a therapeutic option for diabetes and other T cell mediated diseases like MS [115]. Combination vaccination with IL10-DCs and IL-35DCs showed beneficial effects on inhibiting the development of MS. Vaccination induced Treg cells expansion and LAG3 inhibitory molecule expression. However, more studies are needed to determine the best route of administration, cell dosage and the clinical benefit of tolDCs [116].

#### Regulatory T cell (Treg) therapy

Treg cells are the main immune cells with immunomodulatory functions that are involved in the improvement of MS disease. Adaptive transfer of these cells could be a promising treatment approach. Pre-clinical studies on the EAE animal model indicated that Treg cell therapy could inhibit inflammatory responses and decrease the relapse phase. Treg cells also ameliorated the relapse intensity, especially after the disease onset. Antigen-specific Treg cells could migrate to the CNS while constantly expressing FOXP3 and exerting their suppressive activity [117]. Transferred Treg cells reduced the number of inflammatory TCD4^+^ cells and improved the EAE severity and progression [17, 118]. Treg cells comprise a low percentage of blood T cells (5–7%) so, ex-vivo expansion of these cells before cell transfer is necessary. In a recent phase 1b/2a clinical trial autologous Treg cells were administrated in 14 relapsing-remitting MS patients. Freshly isolated autologous CD4 + CD25^hi^CD127^−^FoxP3^+^ Treg cells were injected intrathecally while ex vivo expanded Treg cells were administered intravenously. This trial showed that Treg cell transfer was safe with no severe adverse effects. Moreover, the intrathecal route of administration was found to be more efficient than the intravenous approach [16].

The development of transgenic Treg cells improved Treg therapies. T cell receptor (TCR) specific immunotherapy could target MS specific antigens like MOG with strong affinity and induce constant expression of FOXP3. Engineered CAR/ FOXP3 Treg cells that targeted the MOG antigen showed suppressive effects EAE animal model. CAR Treg cells ameliorated disease symptoms, inhibited inflammatory markers like IFN-γ and impaired DC function [17]. Recently it was demonstrated that dual-specific engineered CAR Treg cells had a better therapeutic efficacy compared to mono-specific CAR Treg cells. MOG/NF-M bi-specific TCR- Tregs which targeted both MOG and neurofilament-medium (NF-M), strongly inhibited inflammatory and autoimmune responses and had a protective effect against inducing EAE with other antigens [119]. Some challenges regarding adaptive Treg cell therapy should be addressed including using additional CD markers to isolate purer Treg cells, generating Tregs with stable FOXP3 expression, cytokine release syndrome (CRS) which usually occurred in CAR-T therapies [15].

Beyond the CAR Tregs, CD19-directed CAR-T cell therapy has emerged as a mechanistically distinct strategy for re-establishing immune tolerance in MS. While CAR-Tregs aim to suppress inflammation through modulation, CD19 CAR-T cells are designed to “purge” the pathogenic B-cells through deep and systemic cellular depletion. Early clinical data from individual treatments and small case series of patients with both progressive and relapsing MS have shown promising results [120, 121]. Current research is expanding into larger Phase 1b trials (e.g., NCT06451159) to systematically characterize the safety and long-term efficacy of agents like KYV-101. Furthermore, investigators are exploring anti-BCMA CAR-T cells to target long-lived plasma cells and modulate pro-inflammatory microglial activation, potentially providing a more comprehensive strategy for halting MS progression [122].

#### Mesenchymal Stromal Cells (MSCs) and Extracellular Vesicles (EVs)

Stem cell therapy is still a broad concept and a promising therapeutic approach that has been recently used in different clinical conditions like autoimmune disorders [123], degenerative diseases [124] and cancers [125]. Adult stem cells (ASCs) are undifferentiated cells that have self-renewal ability and can differentiate into specific cell lines. ASCs categorized into HSCs which are discussed earlier in MSCs. MSCs found in various tissues bone marrow, adipose tissue, Wharton’s jelly, umbilical cord blood, dental and brain tissue. Besides high proliferation rate and differentiation ability, MSCs possess high immunomodulatory and immunosuppressive activity, secret trophic factors, modulate inflammation in damaged sites and, enhance tissue repair and regeneration [126]. Since MSCs express low levels of MHCI molecules they are not immunogenic. Despite the source of MSCs, these cells suppress Th1 and Th17 cell proliferation, inhibit the secretion of inflammatory cytokines, and impede DCs migration and maturation. Moreover, MSCs induce Treg cell proliferation and anti-inflammatory cytokines production. MSCs shift macrophages into the M2 subtype which possesses an anti-inflammatory phenotype (Fig. 2.B) [127]. After infusion, MSCs migrate through disrupted BBB and exert their therapeutical effects. MSCs produce various anti-inflammatory mediators such as TGF-β, IL-10, IL-37, indoleamine2,3-dioxygenase (IDO), prostaglandin E2(PGE2), and growth factors like HGF, vascular endothelial growth factor (VEGF), brain-derived neurotrophic factor (BDNF), NGF, fibroblast growth factor (FGF) which possess neuroprotective and immunosuppressive effects [128, 129]. MSCs could also inhibit immune responses with direct cell-cell interaction through the expression of molecule programmed death 1 (PD-1), induction of FAS-FASL apoptotic pathway and ICOS/ICOSL axis. In addition, MSCs inhibit inflammatory signaling pathways by secreting IL-1ra, TSG6 and PGE2 [130].

Several clinical trials demonstrated the safety, feasibility and, efficacy of MSC therapy on MS patients. The serum levels of IL-10 and IL-4 were increased in MS patients. MSCs could dampen the proliferation and differentiation of Th17 cells and induce Treg cell population. It is supposed that modifying the Treg/TH17 ratio is one of the main mechanisms of transplanted MSCs in improving MS symptoms and re-establishing immune tolerance. In addition, MSC therapy could induce remission, decrease relapse rate and, improve disabilities in treated patients. Safety and efficacy of umbilical cord stem cells (UC-MSCs) in a phase1/II clinical study. It was shown that UC-MSCS were safe and improved MS symptoms like motor function and cognition. Moreover, patients had no new lesions [131]. A recent Phase I clinical trial of allogeneic neural stem cells (NSCs) transfer in progressive multiple sclerosis indicated promising results. For the first time in humans, allogenic NSCs transferred intracerebroventricular in 15 SP-MS patients and showed that NSCs transplantation was safe and clinically stable during 12 months follow up [132]. A randomized double-blind phase II trial (MESMES) assessed safety, tolerability, and efficacy of BM-MSC therapy in 144 active multiple sclerosis in nine countries. Although BM-MSC infusion was safe and well tolerated, no significant improvement for active patients was reported [133]. A phase II clinical trial on 48 progressive MS patients showed that both intravenous (IV) or intrathecal (IT) BM-MSC administration were safe and had favorable effects on limiting disease activity. However, IT administration showed superior effects on decreasing relapse rates. In addition, NEDA status was higher in MSC-IT treated individuals compared with those who received MSC-IV. It is supposed that local administration of MSCs boosted both auto and paracrine therapeutic effects [134].

Recently, MSC derived EVs (MSC-EVs) were recognized as of the main signaling mediators that could decrease inflammatory immune responses while inducing immunosuppressive effects and maintaining peripheral tolerance. MSC-EV containing EP300-interacting inhibitor of differentiation 3 (Eid3) could efficiently block Th17 differentiation. EVs decreased inflammatory cytokines such as IL-12, IL-17, and, IL-6. Moreover, EVs could upregulate anti-inflammatory factors including TGF-β, galactose lectin-1 (Lgals1), and, PD-1. MSC-EVs could induce FOXP3^+^ Treg cells and shift macrophages into M2 phenotype. Due to the circulating nature of EVs, it is considered that MSC-EVs could be an ideal therapeutic tool to induce peripheral tolerance. Some studies attempt to develop engineered MSC-EVs containing specific cargoes to effectively target immune cells [135]. The results of MSC and MSC-EVs therapy in induction of immune tolerance, neurogenesis, and remyelination, indicated that these approaches are safe and feasible. However, multiple factors can affect treatment outcomes such as route of administration, passage and number of transplanted cells, number of injections and, disease status at the time of injection.

Recent studies indicated that EVs derived from neurons and oligodendrocytes could have beneficial effects on EAE pathogenesis through regulating apoptosis. Oligodendrocyte-derived EVs could induce apoptosis in autoreactive T cell clones and reduced neuroinflammation. On the other hand, EVs derived from microglia inhibited apoptosis and improved survival of neurons. Administration of oligodendrocyte-derived EVs in EAE animal model eliminated autoreactive T cells and promoted peripheral tolerance. This intervention regulated immune responses and apoptosis in an antigen-specific manner [136].

## Conclusion

Over the past decade, advances in disease-modifying therapies have substantially improved the management of relapsing multiple sclerosis (RMS), which have resulted in a shift in therapeutic strategies from broad immunosuppression toward durable immune reconstitution and restoration of immune tolerance. Although contemporary therapies effectively suppress relapses and focal inflammatory activity, PIRA and progressive neurodegeneration remain major therapeutic challenges. Moreover, the growing recognition of EBV as a potential upstream driver of immune dysregulation has further strengthened the rationale for therapeutic approaches aimed at restoring immune tolerance.

Based on this paradigm, immune reconstitution therapies seek to induce sustained immunological remodeling through transient immune depletion followed by qualitative immune repopulation. The next generation of precision immunotherapies acts by selectively regulating autoreactive immune responses while preserving protective immunity. These emerging approaches includes: tolerogenic dendritic cells, regulatory T-cell therapies, mesenchymal stromal cells, extracellular vesicles, and other antigen-specific immunotherapies. Although encouraging preclinical and early clinical findings support their therapeutic potential, several challenges remain, including optimization of antigen specificity, long-term safety, biomarker-guided patient selection, scalable manufacturing, and confirmation of durable clinical efficacy in large randomized trials. In conclusion, immune reconstitution and antigen-specific tolerance induction represent complementary therapeutic paradigms that may redefine the long-term management of MS. Continued advances in immunology, biomarker discovery, cellular engineering, and precision medicine are expected to accelerate the translation of these approaches into clinical practice.

## Data Availability

Not applicable.
